# Supplementary material for: Epidemiological research on parent–child conflict in the United States: subgroup variations by place of birth and ethnicity, 2002–2013
Source: PeerJ. 2017 Jan 24;5:e2905. doi: 10.7717/peerj.2905 (PMC5267568; doi:10.7717/peerj.2905)
Supplement: Supplemental Information 1 [file peerj-05-2905-s001.docx]

**SUPPLEMENTAL INFORMATION**

Post-estimation exploratory data analyses were conducted to test the heterogeneity of parent-child conflict across years. To this end, we produced estimates for 2-year intervals (i.e., 2002-2003, 2004-2005, 2006-2007, 2008-2009, 2010-2011, 2012-2013) by ethnic groups. Each paired year of NSDUH data constitutes an independent replication sample. To simplify the presentation of these analyses, we present results in a forest plot format (see figures s1-s4). The chi-square test was used to test for heterogeneity across year intervals. Meta-analyses summarize the replication estimates. The heterogeneity test statistic (115.11, df=5) indicated variations across pairs of years for non-Hispanic Whites (p-value < 0.05). No heterogeneity was identified in the remaining ethnic groups (i.e., non-Hispanic African American, non-Hispanic Asian, and Hispanic).

**--------------------------------------------**

**Insert Figures s1 – s4 here**

**--------------------------------------------**
