# Supplementary figures and images for: Epidemiological research on parent–child conflict in the United States: subgroup variations by place of birth and ethnicity, 2002–2013
Source: PeerJ. 2017 Jan 24;5:e2905. doi: 10.7717/peerj.2905 (PMC5267568; doi:10.7717/peerj.2905)

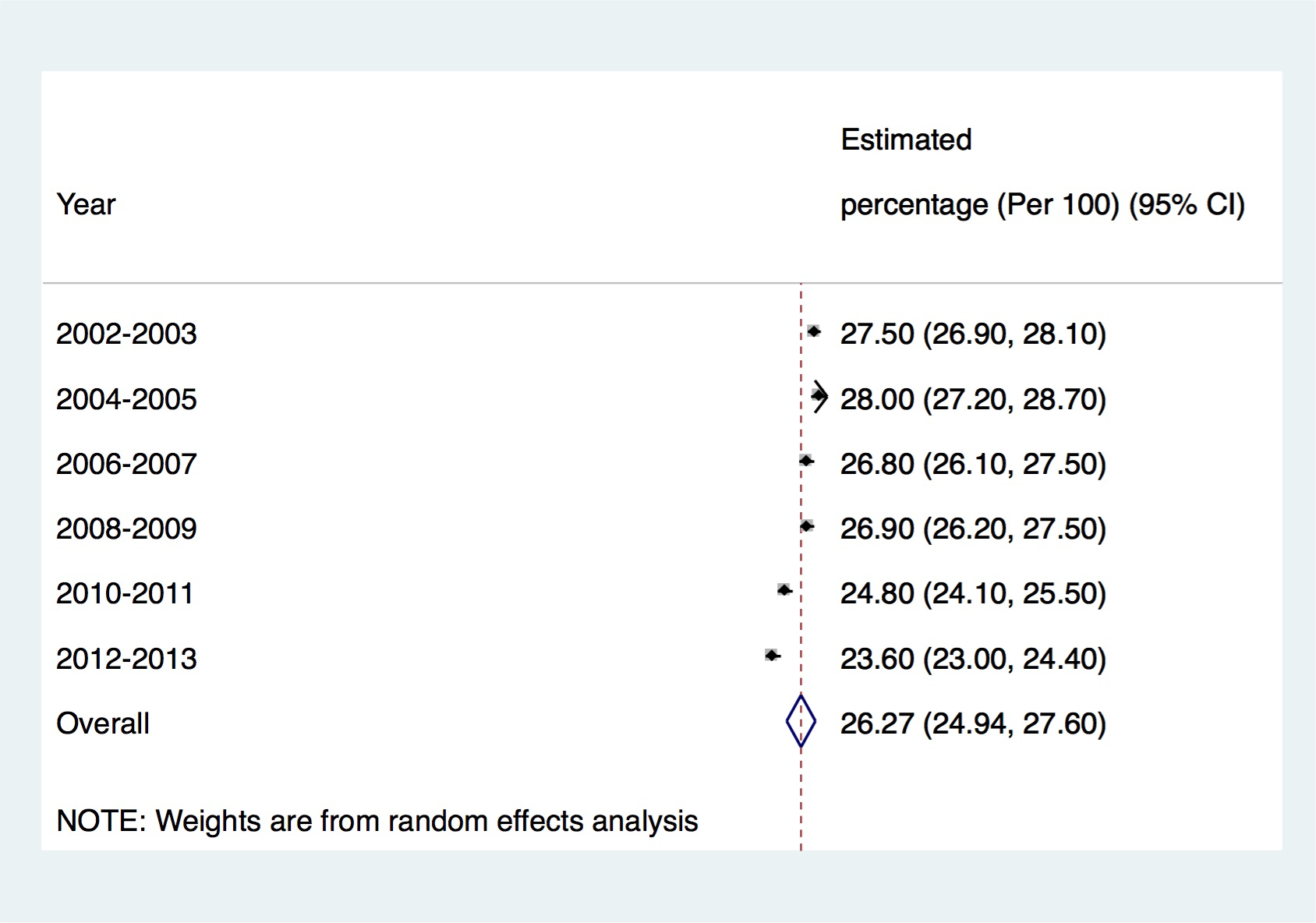

Supplement: Figure S1 [file peerj-05-2905-s002.png]

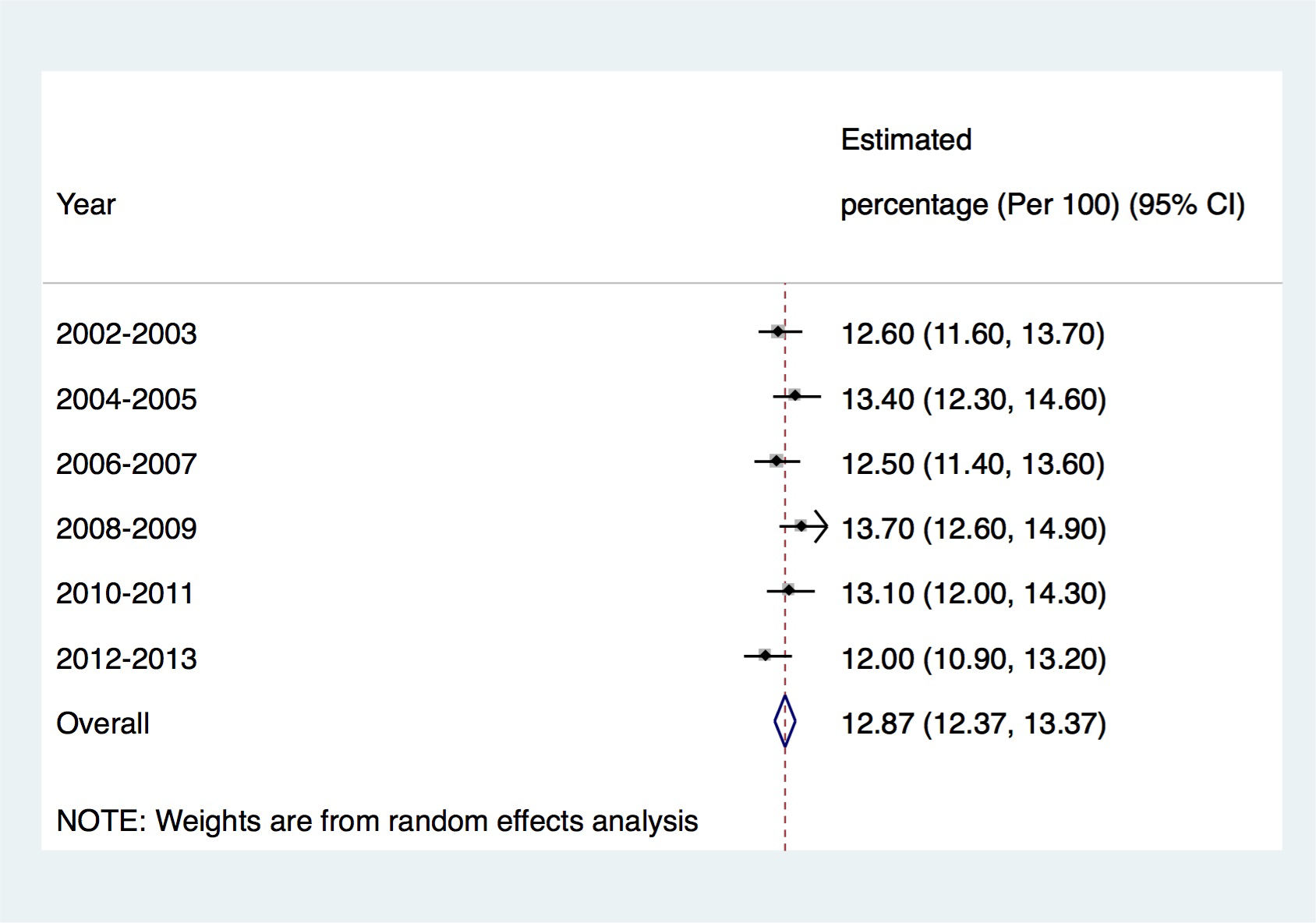

Supplement: Figure S2 [file peerj-05-2905-s003.png]

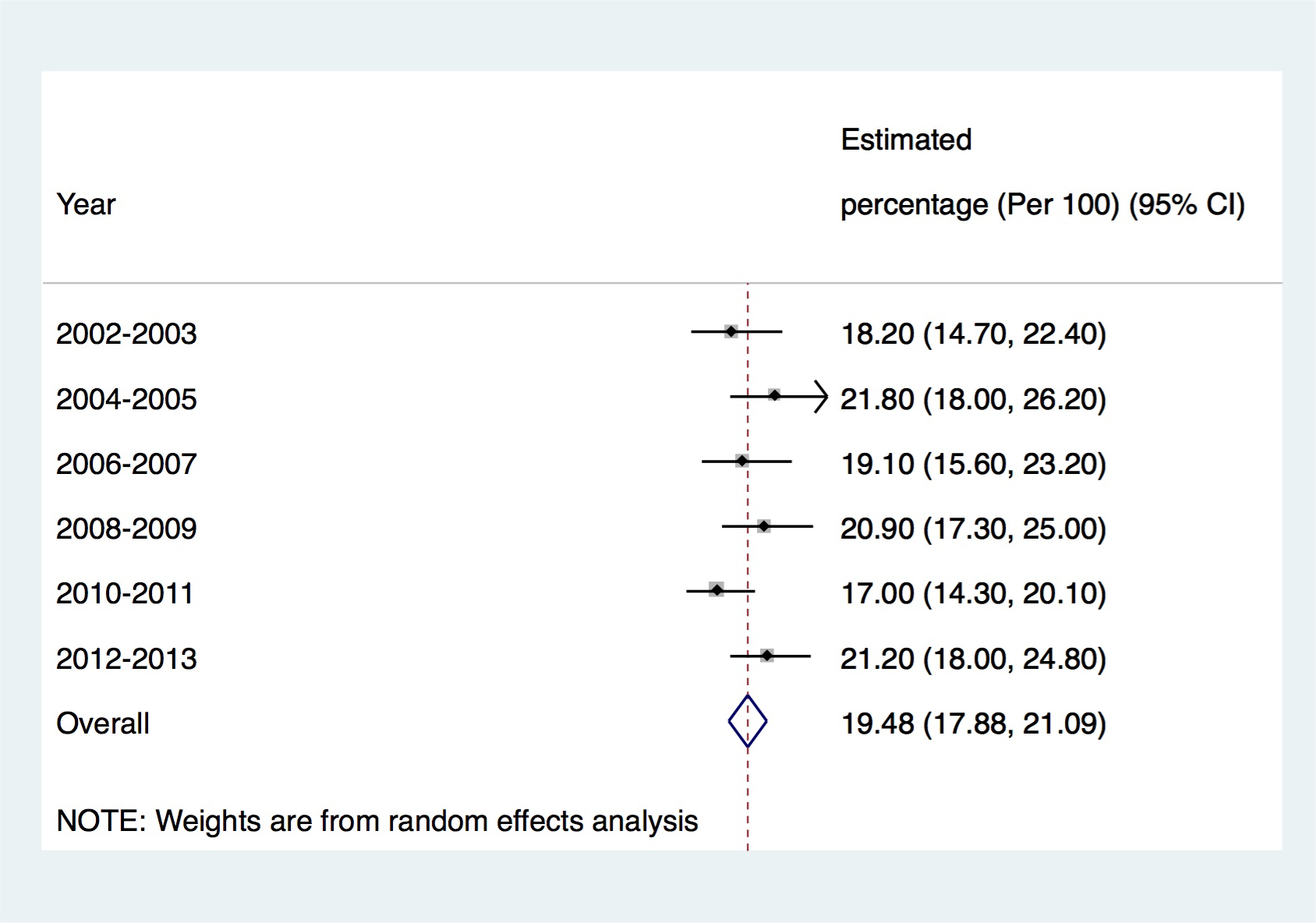

Supplement: Figure S3 [file peerj-05-2905-s004.png]

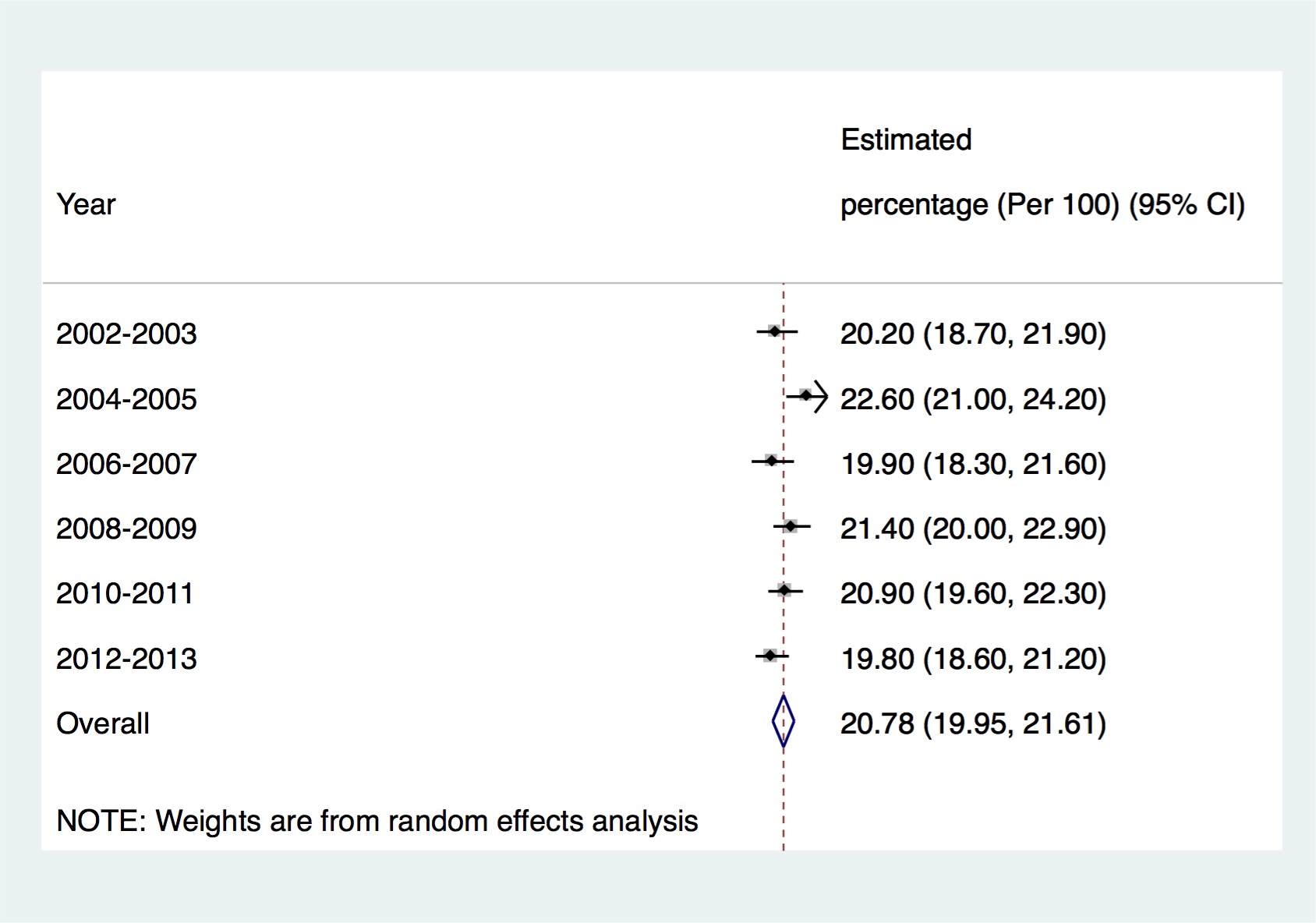

Supplement: Figure S4 [file peerj-05-2905-s005.png]
